# The effectiveness of chronic disease management planning on self-management among patients with diabetes at general practice settings in Australia: a scoping review

**DOI:** 10.1186/s12875-024-02309-4

**Published:** 2024-03-01

**Authors:** Maryam Ghasemiardekani, Georgina Willetts, Kerry Hood, Wendy Cross

**Affiliations:** 1 Institute of Health and Wellbeing. Federation University, Ballarat, Victoria Australia; 2 Nursing and Practice Development Institute of Health and Wellbeing, Federation University, Ballarat, Victoria Australia; 3 Federation University, Ballarat, Victoria Australia

**Keywords:** Chronic disease management plan, Primary healthcare, Self-management, Medicare-rebated CDM plans

## Abstract

**Background:**

Medicare provides significant funding to improve, encourage and coordinate better practices in primary care. Medicare-rebated Chronic Disease Management (CDM) plans are a structured approach to managing chronic diseases in Australia. These chronic disease care plans are intended to be a vehicle to deliver guideline-based / evidence-based care.. However, recommended care is not always provided, and health outcomes are often not achieved. This scoping review aimed to identify the specific components of CDM plans that are most effective in promoting self-management, as well as the factors that may hinder or facilitate the implementation of these plans in general practice settings in Australia.

**Method:**

A comprehensive search was conducted using multiple electronic databases, considering inclusion and exclusion criteria. Two reviewers independently screened the titles and abstracts of the identified studies via Covidence, and the full texts of eligible studies were reviewed for inclusion. A data extraction template was developed based on the Cochrane Effective Practice and Organization of Care Group (EPOC) to classify the intervention methods and study outcomes. A narrative synthesis approach was used to summarize the findings of the included studies. The quality of the included studies was assessed using the JBI Critical Appraisal Checklist.

**Results:**

Seventeen articles were included in the review for analysis and highlighted the effectiveness of CDM plans on improving patient self-management. The findings demonstrated that the implementation of CDM plans can have a positive impact on patient self-management. However, the current approach is geared towards providing care to patients, but there are limited opportunities for patients to engage in their care actively. Furthermore, the focus is often on achieving the outcomes outlined in the CDM plans, which may not necessarily align with the patient’s needs and preferences. The findings highlighted the significance of mutual obligations and responsibilities of team care for patients and healthcare professionals, interprofessional collaborative practice in primary care settings, and regular CDM plan reviews.

**Conclusion:**

Self-management support remains more aligned with a patient-centred collaboration approach and shared decision-making and is yet to be common practice. Identifying influential factors at different levels of patients, healthcare professionals, and services affecting patients’ self-management via CDM plans can be crucial to developing the plans.

**Supplementary Information:**

The online version contains supplementary material available at 10.1186/s12875-024-02309-4.

## Introduction

Chronic disease is the most significant burden on the Australian health system [1]. The Australian government spends $1 billion annually on developing and reviewing chronic disease management and encouraging optimal practice in primary healthcare settings [2]. Optimal health outcomes for chronic disease and reduced risk of complications depend on effective self-management by the individual with chronic disease, and it is essential to ensure healthcare providers facilitate and support sustainable and suitable self-management of chronic diseases such as diabetes on an ongoing basis [3]. Self-management is a practical approach to diabetes care because it empowers individuals to take an active role in managing their condition and reduce the risk of complications associated with diabetes, which can lead to improved decision-making and adherence to treatment plans [4]. Chronic disease management (CDM) is essentially an implementation vehicle to support the delivery of guidelines-based care and is tailored to provide various self-management and tracking systems for people with chronic diseases. The initial enhanced primary care for chronic disease commenced in 1999 and developed significantly for aged care, Aboriginal health, and allied health access in Australia [5]. Medicare is a universal healthcare scheme in Australia that provides,essential coverage of healthcare services for eligible people [6]. The Medicare Benefits Schedule (MBS) subsidises General Practitioner (GP) consultation and some allied healthcare services for a patient with chronic diseases for structural assessment, planning, and multidisciplinary team care under CDM plans. The GP is responsible for initiating a general practice management plan (GPMP), which includes a comprehensive description of the patient’s needs, goals, actions, treatment, service arrangement, and review. Also, to receive ongoing treatment or services through team care arrangements (TCAs), the GP must work collaboratively with at least two other healthcare providers. GPMPs and TCAs (Team Care Arrangements) are initiatives in Australia that aim to enhance the management of chronic diseases. GPMPs are tailored management plans in collaboration with patients to assist them in managing their chronic conditions, and TCAs involve a team-based approach in which a patient’s care is coordinated by a GP in collaboration with other healthcare professionals [5] (Table 1). The relationship between these initiatives is that a CDM plan can include a GPMP and TCA, both of which can be complementary in managing a patient’s chronic disease. Therefore, understanding the interrelation between these initiatives is crucial for both patients and healthcare professionals to manage chronic diseases more effectively The Royal Australian College of General Practitioners [RACGP] [7] recommends preparing a new GPMP and (TCA) every 2 years with a 6-, 12-, and 18-months review. Patients eligible for CDM plans can claim up to five healthcare services provided each calendar year.

**Table 1:** CDM plans components and frequency

Service description	Claiming frequency
Preparation of a GPMP	Once every 12 months
Coordination of the development of TCAs for CDM	Once every 12 months
Contribution to a Multidisciplinary Care Plan or to a review for a patient who isn’t in a residential aged care facility	Once every 3 months

### Rationale

Self-management empowers patients to take an active role in their own care. With CDM plans, patients are equipped with the knowledge and skills necessary to manage their conditions on a daily basis [4]. Additionally, self-management can reduce healthcare costs by minimizing hospitalizations and emergency room visits [8]. MBS summary claims data of 10-year trend analysis between 2006 and 2014 found that more general practitioners (GPs) are utilising the Medicare-rebated CDM items in their practice [9], despite challenges faced by GPs and patients, such as slow uptake and barriers to use [10]. Lack of patient engagement and education, [11], lack of coordination and communication among healthcare professionals and fragmented care [12], failure to tailor the care plan to the specific needs and capabilities of the patient [13] can render the plan ineffective. Additionally, a lack of resources and support [11, 14] can hinder the success of the plan. Finally, a failure to track and monitor patient progress can make it difficult to make necessary adjustments to the plan and ensure its efficacy over time [15]. Additionally, there appears to be a low uptake in CDM plan reviews, highlighting potential gaps in the implementation and follow-up of these plans [16].. There is a lack of evidence regarding the effectiveness of CDM plans in improving patient health outcomes within the current routine of healthcare delivery regarding how these plans are being implemented and what impact they are having on patient outcomes. McCarthy et al. [11] revealed that the available outcome evidence of CDM plans is from single-site trials rather than everyday clinical practice settings. It is essential to know what role healthcare professionals play in supporting people with chronic diseases to enhance their level of functioning and management of their care safely and sufficiently [17]. CDM plans must appropriately target patients’ needs. Nonetheless, current health delivery arrangements of CDM plans such as GPMP and TCA often poorly serve patients with chronic conditions as they fail to adequately coordinate care across different service providers and care settings [11].

Moreover, there is growing evidence of the impact of allied health interventions for chronic diseases such as diabetes and cardiac and respiratory diseases [9, 18–20]. Given the complexity of managing people with different chronic conditions, potential interventions will likely be complex and multifaceted if they address these individuals’ varied needs [21, 22]. A variety of healthcare professionals will be involved and collaborate in the multidisciplinary team and with the patients in the interactive platform via CDM plans to share information about patients promptly to achieve better health outcomes. There are gaps between patients’ needs and what is available or provided. Understanding why some patients have poor self-management would support healthcare providers in offering person-centred, well-organized, and appropriate guides through CDM plans and improving the healthcare delivery system. This scoping review is important for healthcare providers because of the potential to identify the barriers and determine the strengths and weaknesses of CDM planning and what they and their organizations could do to increase better health outcomes. The gaps mentioned previously may affect decision-making about appropriate allied health involvement resulting in a mismatch of care provision with patients’ needs. In addition, some patient-driven motivators might influence CDM plans’ appropriateness. To conclude, there is a lack of evidence to support the impact of CDM plans on health outcomes and a significant need for CDM plans that improve access to allied health services to improve patients’ self-management and the efficiency of care delivery.

### Research questions

The primary research question of this scoping review is: “To what extent do CDM plans facilitate self-management support for T2DM?”

This scoping review systematically examined the scope and characteristics of the research on the topic. More precisely, this scoping review discusses 1) the extent (the amount or quantity of evidence), range (the range of evidence on CDM plans might include studies conducted with different populations, and using different methods), and nature (strength of the study designs used, the size of the study samples, and the consistency of the findings across different studies) of the evidence on the topic, 2) summarises the main findings from existing research, and 3) identifies gaps in the research to recommend and inform future research on CDM plans on patients’ self-management.

## Method

A scoping review was considered suitable for this review as this method systematically identifies and maps from wide-ranging available evidence [23]. To enable rigorous review, the 22-item Scoping Review Checklist (SRC) was applied [24]. Also, this scoping review included the methodological guidance of the JBI [25]. The approach was selected as it allows systematic exploration of a complex and multivariable topic, identifying gaps in knowledge and research activity [25].

The effectiveness of CDM plans on patient self-management and overall patient health outcomes was analysed in our scoping review methodology. The data analysis process was iterative and involved reworking and refining our research questions as our understanding of the data matured. The first analysis stage consisted of a descriptive analysis, where methods from thematic analysis were used [26]. Broad questions were asked, such as: What interventions were used? With what goal? For whom? For how long? How was it measured? Each paper was analysed again using methods from descriptive thematic analysis, such as identifying defining characteristics and attributes, modelling and contrasting cases, antecedents, and consequences [27]. During the analysis, it was noticed that studies seemed to differ based on underlying ideas about the role, place, and value that CDM plans have (or should have) in patient self-management. A decision was made to carry out a third analysis, aiming to identify and map how CDM plans were discursively positioned regarding patient self-management [28]. The realities of implementing and executing CDM plans and how they may enable, or hinder patient self-management were sought to be understood through this process. Each article’s introduction, literature review, and discussion were analysed for its positioning of CDM plans to patient self-management or overall patient health outcomes, focusing on prominent discourses and their associated rationales and authorities [29]. Guiding analytical questions were: What relationship between CDM plans and patient self-management is constructed in the sample? What contexts are in which the value of CDM plans for patient self-management is constructed?

Due to the diverse findings, data analysis, synthesis, and reporting were achieved using the PRISMA Extension for Scoping Reviews (PRISMA-ScR).

### Data sources

A systematic search of the databases, including CINAHL, EBSCO, OVID, MEDLINE, BMJ, EMBASE, PUBMED, the Cochrane Library, PsychiNFO, Science Direct,,andWiley Online Library was undertaken in May 2022 (Table 2), and Appendix 1 is full search strategy for all databases. These search terms can be combined using Boolean operators such as “AND” or “OR” to retrieve relevant articles from databases. Sources were limited to those in English, peer-reviewed, and published in 2012–2022, confirming current research and nursing practice. As recommended by the JBI [25], various searches for the grey literature were also conducted. In our search for relevant literature, we included relevant grey literature databases such as Grey Literature Report, Open Grey, and Google Scholar. We identified studies relevant to this review by using specific keywords and search terms related to the topic of our study. Additionally, we also scanned the reference lists of the articles we found to identify any other relevant studies that we might have missed in our initial search. This methodology ensured that our search for literature was comprehensive and replicable [30].

**Table 2:** Databases used to search for relevant literature

Database	Results
MEDLINE	89
CINAHL	82
PUBMED	76
EMBASE	23
Science Direct	10
Wiley Online Library	30
BMJ	39
Google Scholar	113
EBSCO	28
OVID	34
Cochrane Library	15
PshychiNFO	26
Grey Literature	12
Total number of articles	577

### Study selection

**Fig. 1:**
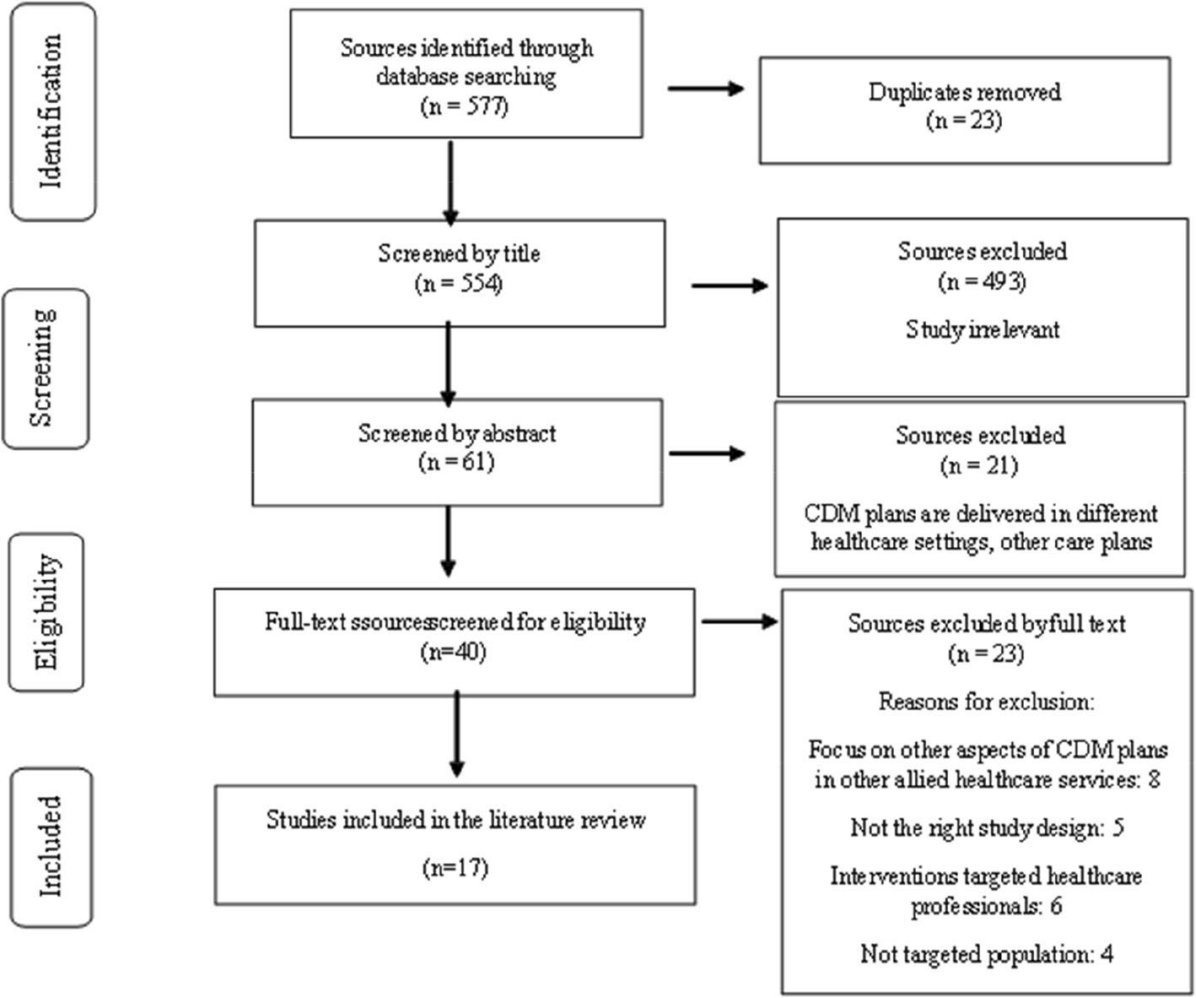
PRISMA flowchart of the search strategy

All studies were manually imported to Covidence systematic review software (Covidence, Veritas Health Innovation, Melbourne, Australia).. Studies (n = 577) were screened for eligibility using pre-determined inclusion and exclusion criteria (Table 3). Some duplicates (*n* = 23) were removed automatically by Covidence [31]. Thereafter, the first author (MG) verified duplication accuracy from the review. The initial database search results were also screened by the first author (MG) using title (*n* = 554) and then abstract (*n* = 89) screening for eligibility. The full-text article was reviewed if the abstract was unavailable or where eligibility could not be determined. The reference list of the articles was reviewed for further relevant publications (MG). Two reviewers (GW and MG) then reviewed and screened the full-text articles to ensure that the inclusion criteria were met. One independent reviewer (WMC) resolved any conflicts (Fig. 1, the PRISMA flowchart).

**Table 3:** Inclusion and exclusion criteria

Inclusion criteria	Exclusion Criteria:
**Population:** • Patients with diabetes type 1 or 2 who are eligible for CDM plans or patients with other common chronic diseases such as chronic obstructive pulmonary disease and obesity • Healthcare professionals involved in the CDM plans. (Healthcare professionals involved in the CDM plans related to diabetes management including, GPs, practice nurses, dietician, diabetes educators, exercise physiotherapist, and podiatrist)	**Population:** **•** Participants aged under 18 years. • Healthcare professionals not related to CDM plans
**Concept:** • Delivery of CDM plans, specific CDM plans rebate by Medicare in Australia • Reported patients’ or healthcare professionals’ perceptions related to self-management support in CDM plan encounters, including goal setting, person-centred care, shared decision-making, and patient-provider interactions	**Concept:** • Other care plans • Reported patient or healthcare professionals’ perceptions/experiences of self-management unrelated to the primary healthcare settings
**Context:** Australian Health outcomes related to CDM plans	**Context:** Education provided in other healthcare settings, different health outcomes
**Settings:** General practice settings and community-based healthcare services	**Setting:** Other healthcare settings, such as inpatient, residential, aged care, or palliative settings

Although some literature included a range of CDM plans with other health conditions, patients with diabetes were either a subgroup or the focused population of all included sources. Disagreements between reviewers were discussed, and agreement was attained.

### Data extraction and elements of CDM plans

A data extraction template was developed based on the Cochrane Effective Practice and Organization of Care Group (EPOC) to classify the intervention methods and study outcomes [32]. Using methods developed for previous reviews [33, 34], two reviewers (MG and GW) completed data extraction. Appendix 2 is the data extraction form that was developed in Covidence. In the context of EPOC, the elements related to the effectiveness of chronic disease management plans on patient self-management can be categorized according to the type of intervention used. Some common elements that may be relevant to EPOC include 1) Organizational interventions: implementing changes to the organization and delivery of healthcare services, such as providing patient-centred care or improving care coordination, can help to support patients in their self-management efforts. 2) Financial interventions: providing financial incentives or removing financial barriers to accessing healthcare services can help to improve patient engagement and self-management. 3) Regulatory interventions: implementing regulations or guidelines that promote patient-centred care and self-management can help to improve the quality of care provided. Framework. Any discrepancy was resolved by an independent reviewer (WMC). The elements included self-management support, the effectiveness of the CDM plan on long-term and short-term outcomes, and overall enhanced person-centred care outcomes to meet the needs of patients and healthcare professionals.

### Charting the data

Data were charted according to the included studies’ aims, objectives, methods, location, sample, and key findings. The location was characterized by the country of origin and general practice settings where the research was conducted. The study methods were charted based on the design, data collection, and analysis.

### Quality appraisal

Forty articles were subjected to a final full-text review to ensure minimum research criteria were met. This suggests that all 40 articles were screened in their entirety to determine if they met the inclusion criteria for the review, which likely included factors such as study design, sample size, and methodology. But only 17 of them met the inclusion criteria for review. The remaining 23 articles were excluded from the review for various reasons. The quality appraisal process was performed on all 17 articles to ensure that the studies included in the review were of high quality and that the results could be relied upon [25]. Assessing aspects of the research, including design, recruitment, data collection, ethics, the rigor of the data analysis, results, and the significance of the study to practice, were examined. Three authors (MG, GW, and KH) were also involved in bias appraisal. All authors independently assessed the bias risk of the 40 articles. The JBI critical appraisal tool was used to identify elements related to CDM plans and the effectiveness of CDM plans in improving health outcomes such as self-management [25, 27] (See Table 4). Each question is scored as “yes,” “no,” or “unclear,” and the total score is presented as a percentage. A score of 50% or less is considered weak, 50–75% moderate, and over 75% strong. The risk of bias is also assessed, and it involves identifying any factors that may have influenced the study results, such as selection, performance, detection, attrition, and reporting biases [25]. Seventeen articles were included in the review for analysis and thematic integration of the research findings (See Fig. 1). Any discrepancies were adjudicated by the fourth author (WMC).

**Table 4:** Quality analysis using JBI critical appraisal tools

JBI Critical Appraisal Checklist for Systematic Reviews and Research Synthesis															
Author(s)	Q1	Q2	Q3	Q4	Q5	Q6	Q7	Q8	Q9	Q10	Q11			Total	Risk^b^
Davidson et al. (2022)	✓	✓	✓	✓	✓	✓	?	✓	?	✓	✓			81%	Low
Franklin et al. (2018)	✓	✓	✓	✓	✓	×	×	✓	×	✓	✓			72%	Low
Reynold et al. (2018)	✓	✓	✓	✓	✓	✓	?	✓	×	✓	✓			72%	Low
JBI Critical Appraisal Checklist for Cohort Studies															
Author(s)	Q1	Q2	Q3	Q4	Q5	Q6	Q7	Q8	Q9	Q10	Q11			Total	Risk^b^
John et al. (2020)	✓	✓	?	✓	✓	?	✓	✓	✓	×	✓			72%	Low
Welberry ^b^ et al. (2019)	✓	✓	?	✓	?	✓	✓	✓	✓	?	✓			72%	Low
Barr et al. (2019)	✓	✓	?	✓	×	✓	?	✓	✓	?	✓			63%	Moderate
JBI Critical Appraisal Checklist for Analytical Cross-sectional Studies															
Author(s)	Q1	Q2	Q3	Q4	Q5	Q6	Q7	Q8						Total	Risk^b^
Welberry ^a^ et al. (2019)	✓	✓	✓	✓	?	?	✓	✓						75%	Low
JBI Critical Appraisal Checklist for Case study															
Author(s)	Q1	Q2	Q3	Q4	Q5	Q6	Q7	Q8	Q9	Q10				Total	Risk^b^
Choi et al. (2017)	✓	✓	?	✓	✓	✓	?	✓	✓	✓				80%	Low
JBI Critical Appraisal Checklist for Randomized Controlled Trials															
Author(s)	Q1	Q2	Q3	Q4	Q5	Q6	Q7	Q8	Q9	Q10	Q11	Q12	Q13	Total	Risk^b^
Coorey et al. (2022)	✓	?	✓	?	?	?	✓	✓	✓	✓	✓	✓	?	61%	Moderate
JBI Critical Appraisal Checklist for quasi-experimental studies															
Author(s)	Q1	Q2	Q3	Q4	Q5	Q6	Q7	Q8	Q9					Total	Risk^b^
Wickramasinghe et al. (2013)	✓	✓	✓	×	✓	✓	?	✓	✓					77%	Low
JBI Critical Appraisal Checklist for Qualitative Research															
Author(s)	Q1	Q2	Q3	Q4	Q5	Q6	Q7	Q8	Q9	Q10				Total	Risk^b^
Hegney et al. (2013)	×	✓	✓	?	✓	?	?	✓	✓	✓				60%	Moderate
Vasi et al. (2020)	×	✓	✓	✓	✓	?	?	✓	✓	✓				70%	Low
Holden et al. (2012)	×	✓	✓	?	✓	?	?	✓	?	✓				50%	Moderate
Khoo et al. (2019)	×	✓	?	?	✓	?	×	✓	✓	✓				50%	Moderate
Kennedy et al. (2021)	×	✓	✓	✓	✓	?	?	✓	✓	✓				70%	Low
Fuller et al. (2015)	×	?	✓	✓	?	?	×	?	✓	✓				40%	High
Foster and Mitchell (2013)	×	✓	✓	✓	✓	?	×	✓	✓	✓				70%	Low

The risk of bias was ranked as high when the study reached up to 49% of “yes” scores, moderate when the study reached from 50 to 69% of “yes” scores, and low when the study reached more than 70% of “yes” scores. ‘✓’ indicates yes, ‘✕’ indicates no and ‘?’ indicates unclear.

### Synthesising and reporting the data

A narrative synthesis was considered to summarise and explain the results. The review was directed by key essentials of the narrative synthesis framework by Popay et al. [35]. The data was grouped, tabulated, and analysed thematically [28] based on the following categories: 1) General information including the author, year, and type of review was considered. 2) self-management measurements including information on how self-management or other health outcomes were measured in the studies was tabulated. 3) The data was categorized into four broad categories that included behavioural changes and making lifestyle changes, challenges of self-management support via CDM plans, communication with healthcare providers, and navigation through the healthcare system. 4) All types of information concerning the relationship between CDM plans and self-management were analysed thematically to identify themes related to the types of self-management support provided, the challenges encountered in self-management, the effectiveness of different interventions, and the barriers and facilitators to patient self-management.

The final step was examining the sources’ strength and assessing the evidence from which conclusions and generalizations would be made [35].

## Results

### Descriptive findings

The final data set entailed two case studies, one cohort, one cross-sectional, one integrative review, two prospective longitudinal studies, one before and after study of perspective collected data, one mixed method, one secondary analysis of baseline data from the connected randomized controlled trial, one secondary analysis of qualitative data, four qualitative research, and two systematic reviews. The combination of sources addressed three different scopes of the CDM plan: Factors that influence patient adherence (*n* = 5), healthcare professional services (*n* = 5), and system management (*n* = 7). Primary and community health settings were considered, including primary care, family practice, and community-based care delivered by healthcare professionals, including general practitioners, practice nurses, and allied healthcare professionals directly involved in CDM plans. Three studies focused on the provision of care for diabetes [13, 36, 37], one study focused on cardiovascular diseases [38], and the remaining studies focused on the provision of care for overall chronic conditions [9, 19, 20, 33, 38–44]. Study participants included patient and nurse encounters during a care plan application [13, 45], patients with chronic diseases [9, 19, 20, 36, 39, 40], and healthcare professionals [37, 41–44]. The objectives of all articles focussed on self-management person-centered care, shared goal settings, availability and accessibility of services through CDM plans, system constraints, and the interprofessional collaboration between the multidisciplinary team involved in the CDM plans (Table 5). It needs to be noted that all the studies included in this review were conducted in Australia. This is because the model of CDM used in Australia is distinctive from the subsidized Medicare model within the Australian healthcare system. Therefore, it is important to understand how CDM plans are implemented and their effectiveness within the context of the Australian healthcare system. Furthermore, healthcare systems can differ significantly between countries, and what works in one country may not work in another. By limiting the study to only those conducted in Australia, can be assured that the findings are relevant and applicable to the Australian healthcare context with similar regulatory and cultural environments. This can help to reduce the potential for bias and confounding factors that can arise when comparing studies conducted in different countries with different healthcare systems.

**Table 5:** Summary of included studies: examining use of CDM plans related to diabetes self-management in the Australian context

Author/Date	Aims/ Objectives/ Research Questions	Setting	Participants	Design/Method	Key finding
[46]	To give practice nurses a greater focus on prevention and education on chronic disease management.	9 general practices in southeast Queensland	26 healthcare professionals	Exploratory qualitative research	Time pressures and unreliable MBS information were barriers. Employing a nurse, team-based approaches, recall systems, and using only selected MBS CDM item numbers were enablers to uptake for general practitioners
[43]	To explore the perceptions and experiences of the staff and patients with Nurse-led CDM plans	Primary healthcare settings (Queensland)	A total of 3 PMs, 5 Nurses 5 GPs, patients (*n* = 38)	A concurrent mixed-method study was situated within the paradigm of Pragmatism	The collaborative involvement of doctors was an intrinsic factor in patient acceptability of nurse-led care that facilitated positive outcomes for nurses. Chronic disease management delivered by nurses was acceptable, feasible, and sustainable.
[36]	To investigate whether GPMPs and TCAs, and reviews improve the management and outcomes of patients with diabetes by cdmNet	Patients with type 1 or 2diabetes mellitus from across Australia (including metropolitan, rural and regional communities	Patients with type 1 or 2 diabetes mellitus (*n* = 577)	A before-and-after study of prospectively collected data	There were significant improvements in process and clinical outcomes for patients on a GPMP or a GPMP and TCA with regular review and no significant change without reviews
[39]	To investigate the perspectives of primary care patients in receipt of Medicare-funded team care for CDM	Two purposively selected general practices: one urban and one regional practice in Queensland	Patients (n = 23)	Qualitative study	If there is a sense of personal obligation and sufficient financial incentive, and considering patient expectations and preferences, patients are likely to engage with a structured team care approach to CDM. and decision
[42]	To draw on the implementation experience of the South Australian GP Plus Practice Nurse Initiative for developing the chronic disease management role of practice nurses.	147 General Practices in Adelaide	Three focus groups: 41 practice nurses and one group: 10 practice nurse coordinators and practice nurse mentors.	Secondary analysis of qualitative data contained in the Initiative evaluation report. (GP Plus Practice Nurse Initiative Final Evaluation Report 2007—2010)	Support is needed at two levels to advance the role of practice nurses as managers of chronic disease and to assist practice nurses in building their skills. Support is also needed to ensure that systems are ready to include the practice nurse within the practice team
[13]	To understand Chinese migrants living with T2DM experiences in Australia and their culturally specific diabetes management needs, habits, and expectations in the	GP clinics (two Melbourne and one Sydney)	Patients/providers (*n* = 18/8)	Case study	Chinese migrants look to their peers more for diabetes management because healthcare professionals are not part of supporting the community. Also, redesigning diabetes management services align with collectivism which is appropriately much with patient’s expectation
[33]	To review studies investigating the experience of self-management support in patient and provider interactions and shaping goals.			Systematic review and qualitative synthesis	Interactions are affected by consultation times, patient self-blame and guilt, desire for autonomy, and beliefs about what constitutes practical self-management skills. Healthcare professionals remain in a position of authority because of limiting opportunities for control to be shared with patients and shared understandings of social context
[34]	To evaluate health outcomes in chronic disease management interventions for adults with chronic diseases implemented in primary or community care settings			Systematic review with narrative synthesis	Self-Management support is the most frequent Chronic Care Model intervention associated with significant improvements statistically and for diabetes and hypertension, predominately
[19]	To examine utilization rates of GPMPs or TCAs, characteristics, and relationship with hospitalization for cohort participants of Central East Sydney over the period 2006–2014	Primary and Community Health Cohort/Linkage Resources (Central and Eastern Sydney)	Patients with chronic disease (*n* = 30,645)	A cross-sectional study	Well-targeted GPMPs and TCAs in the CES area with no relationship with prevented hospitalizations in the CES region.
[20]	To describe the characteristics of people in Central and Eastern Sydney (CES), NSW, who had a (GPMP) and claimed for at least one private allied health service and its relationship with fewer hospitalizations over 5 years.	NSW Centre for Health Record Linkage	Patients (*n* = 5771)	Prospective longitudinal study	Well-targeted usage of allied healthcare. Physiotherapy services were associated with less avoidable hospitalizations.
[9]	To examine person the proportion of claims for preparation and review of GPMPs or TCAs and allied health services in New South Wales (NSW) by demographic features, chronic conditions, and levels of disability between 2006 and 2014 for any change in uptake	NSW Centre for Health Record Linkage (CHeReL)	Patients (*n* = 264,732)	Longitudinal study	Increasing usage of care plans and allied health services. Increasing care plan reviews, but with suboptimal proportions, may indicate poor continuity of care. .
[41]	To explore the current activities of a sample of Australian private health insurance (PHI) funds to support the care of people with chronic conditions and a permitted change for a broader range of chronic disease management (CDM) services.	PHI sector and hold a senior management role. Invitations to participate were sent via email to 19 PHI organisations (Sydney)	10 Senior management role	Qualitative	After 10 years, insurers are still in the early stage of implementing and evaluating CDM activities, with the primary category of activities in health navigation, disease management, and health coaching programs and care coordination services. Challenges and constraints with patients and other healthcare services and stakeholders were investigated
[45]	Evaluate clinical outcomes after standard care between baseline and 12 months and assess changes in participants’ self-reported HR-QoL, risk of hospital admissions, disease-specific risk, and explore predictors of treatment uptake, response, and compliance	Primary care practice (Sydney)	Patients with diabetes (*n* = 589 and 7750 in the comparison group)	A cohort study design with a comparison group and a case-series study design	Self-management behaviours, the baseline lifestyle, and other health behaviours of the sample participants recorded at the start of the program enable GPs to understand individual needs and quality care better.
[44]	To explore the effectiveness of cdmNet (an eHealth tool) for chronic disease management in general practice settings	database of 800 General practices (metropolitan Melbourne)	34 clinical and non-clinical staff	Qualitative case study	Changes in clinical and organizational routines, team-based approach, allocating resources, training, and supportive leadership can support a structured CDM approach for health innovations
[40]	To explore patients’ experience with chronic conditions in interprofessional collaborative practice in primary care			Integrative review	Three themes were developed: Interacting with healthcare teams, valuing convenient healthcare, and engaging in self-care with an emphasis on patients’ interprofessional collaborative practice
[37]	To explore the perspectives of healthcare staff delivering care to people with diabetes regarding an existing healthcare service	community health service in regional Victoria	21 Healthcare professionals	Qualitative	A more integrated, team-focused, and accessible Model of care (MoC) is needed in a regional area for better outcomes, and the barriers were investigated
[38]	Secondary analysis of baseline data from the CONNECT randomized controlled trial linked to Medicare Benefits Schedule (MBS) and Pharmaceutical Benefits Scheme (PBS) claims.	Twenty-four primary care services in Sydney, Australia. Study sites were spread across greater Sydney, including the Blue Mountains region, and one service was an Aboriginal Community Controlled Health Service.	905 trial participants from 24 primary health care services	Secondary analysis	The risk of CVD in people with or at elevated GPMPs is under-utilized overall. Well-target high-needs populations and facilitated allied health access but without associated with improved CVD risk management

*T2DM* Type 2 Diabetes Mellitus: *HR-QoL* Health-Related, Quality of Life: *GP* General Practitioner: *GPMP* General Practitioner Management Plan: *TCA* Team Care Arrangement: *CES* Central East Sydney: *NSW* New South Wales: *MBS* Medicare Benefits Schedule: *eHealth* Electronic Health: *PHI* Private Health Insurance: *MoC* Model of Care: *PBS* Pharmaceutical Benefits Scheme: *CVD* Cardiovascular Disease

### Synthesis of findings

Most articles in this review highlighted the value of using CDM plans to improve patient self-management. A patient-centred care paradigm was evident with two emerging themes: Limited opportunity for patients to engage and CDM plan outcomes.

#### Barriers to patient engagement in CDM plans

It was challenging to incorporate psychosocial aspects of self-management into the goal-setting process without discussing it. The psychosocial impacts of living with a chronic condition were rarely considered in interactions between patients and healthcare professionals. These issues might not be a priority for healthcare professionals. Furthermore, patients were reluctant to raise their issues due to fear of judgment, lack of rapport, and trust due to time pressure [33]. There was evidence of limitations in patient engagement because of a lack of motivation [33], lack of knowledge and confidence [45], language barriers, and lack of cultural understanding [13, 33]. Mutual obligations and benefits of team care should be framed in both responsibilities of patients and healthcare professionals. Interprofessional collaborative practice in primary care could increase engagement in self-care [40]. In an integrative review, Davidson et al. [47] reported that, across different healthcare settings and conditions, patients consistently wish to be seen as a person rather than to be labelled as a disease. Interaction with the healthcare team was identified as important to patients in terms of looking beyond the condition and being seen as an individual. However, the dominant view was that patients hold the responsibility for self-management, and the onus remains on patients [33, 39].

#### CDM plan outcomes

##### Overall positive changes

Many studies demonstrated substantial positive changes for health professionals and patients. At the health professional level, these included an extended network of health providers [39], collaborative relationships [43], trusting and long-lasting relationships [33] and a holistic approach to care [37].

At the patient level, positive outcomes included convenience of care, shared time and space, and affordability [40]; structured disease management [41]; improved knowledge of the disease and risk behaviour [34]; increased access to healthcare services and care monitoring [46]; individualized assessment and action plans, follow-up, and coordination [48].

##### Clinical changes

After creating GPMPs, one study reported total cholesterol level, low-density lipoprotein (LDL), and body mass index (BMI) significantly improved, and the application of GPMP and TCA improved patient glycated haemoglobin (HbA_1C_) levels [36]. In contrast, a GPMP was not associated with positive outcomes such as improved adherence and clinical targets according to guidelines recommended for cardiovascular disease [38].

The distribution of healthcare services usage via CDM plan:

Factors associated with high GPMP usage included older age, lower education, lower household income, or comorbidities such as diabetes, having a healthcare card, more severe physical limitations, comorbidities, and disabilities [20, 38]. Podiatry and physiotherapy claims were the highest among allied health services over time [19, 20].

##### The importance of CDM plan review

For people with chronic health conditions, Medicare subsidizes structured assessment, planning, and multidisciplinary care under the chronic disease management plan initiative [5]. Within this initiative, a GP can initiate a review of either GPMP or TCA once every 3 months. Medicare-rebated CDM plans support GPs to claim for a maximum of one GPMP preparation and one TCA every 12 months, with the GPMP review at six, 12, and 18 months [5]. CDM plan review is an essential aspect of managing chronic conditions in primary care settings. The review involves evaluating a patient’s progress against their CDM plan, identifying any changes in their condition, and updating their plan accordingly. While CDM plan review is a crucial element of effective chronic disease management, evidence suggests that its usage is much lower than that of the GPMP and TCA initiatives [9, 38] Significant clinical improvement was achieved in patients with regular reviews compared with no reviews [36]. Updating disease management plans was feasible with a regular review via the WellNet program, a patient-centred medical home [45], which evaluated the program’s effectiveness in improving clinical outcomes during follow-up, such as blood pressure among primary care patients.

##### Long-term outcomes

Only two studies assessed long-term outcomes such as hospitalization. According to these studies, there was no statistically significant difference in the rate of emergency and preventable hospitalization over a five-year period between patients who had GPMP and TCA and those who did not have these plans [19]. However, Barr et al. [20] found no statistically significant differences in the rate of potentially preventable hospitalization over 5 years between patients who had five or more physiotherapy claims and those who had no claims. There were no statistically significant differences in hospitalization rates between other allied health service provisions and patients who did not receive these services.

## Discussion and conclusion

### Discussion

Current CDM plans have limited effect on patients’ self-management for a multitude of reasons. It was found that the information on CDM plans lacked detail, specifically about their primary purpose, condition, clinical data, allied healthcare services used, number of sessions, frequency, and specific health outcomes for certain health conditions like diabetes. As a result, it was difficult to create a comprehensive narrative of the issues and determine what improvements were necessary to enhance CDM plans for self-management support. A preliminary finding of the study is that there is substantial variability in the way CDM plans are developed and implemented in primary care settings. This variability may be due to differences in healthcare policies, funding models, and organizational structures. As a result, it is challenging to draw definitive conclusions about the effectiveness of CDM plans in improving patient self-management. However, despite this variability, there is evidence to suggest that CDM plans can be effective in improving patient self-management when implemented correctly. The study highlights the need for a more standardized approach to developing and implementing CDM plans in primary care settings. This approach should consider the unique needs and circumstances of individual patients while also ensuring consistency and accountability across different healthcare organizations and settings. To reach this approach, the need to consider all determinants and improve the health system within general practice setting to promote the uptake of CDM plans. Addressing patient determinants such as language and communication barriers [13, 20] health beliefs, and social factors [33] and healthcare professional determinants such as experience level, training, and collaboration [42, 43, 46] can lead to better patient outcomes and more effective management of chronic conditions.

CDM plans are designed to help individuals with long-term health conditions manage their health effectively. However, the direct link between CDM plans and health outcomes, such as optimal clinical findings, was unclear. A study comparing outcomes from the implementation of different methods of CDM delivery with standard care found no significant variations in outcomes such as BMI, weight, and lab results [45]. Similarly, the lack of regular CDM plan review was considered for not improving clinical findings for patients with diabetes [36] and for patients with cardiovascular diseases [38]. This suggests that simply having a CDM plan in place may not be enough to improve clinical outcomes and that there may be other factors at play. These findings suggest that there may be limitations to the effectiveness of current CDM plans in achieving their intended goals. Regarding long-term outcomes such as emergency and potentially preventable hospitalization, there were no significant differences between having GPMP and TCA in the subsequent 5 years in Central and Eastern Sydney [19]. A time series analysis of MBS CDM claim in New South Wales (NSW) between 2006 and 2014 showed increased initial plan and plan review over time [9]. However, increased CDM plans utilization and review are still at much lower rates than overall GPMP, and there is no evidence of their effects for both studies.

The distribution of CDM plans for better-targeted services based on patients’ needs is another challenge to the effectiveness of CDM plans. If all patients with diabetes are given the same CDM plan, regardless of their specific needs, the plan may not be as effective as it could be. To address this challenge, healthcare providers need to develop more personalized and targeted CDM plans that are tailored to the individual needs of each patient. This may require additional resources and efforts to identify patients’ needs and develop customized plans. The study by Barr et al. [20] on MBS CDM claims in NSW between 2006 and 2014 reported that podiatry in older ages and physiotherapy in younger ages had the highest rates of allied health services utilization, and there was an association between physiotherapy services and reduction in hospitalization. However, the use of physiotherapy services might be related to unmeasured aspects of a patient’s health status, which is unclear in the study. There was no association between hospitalization and other allied health services usage such as dietician, diabetes educator, podiatrist, etc. Similarly, evaluation of the utilization of GPMP in patients with or at elevated risk of cardiovascular disease demonstrated no association between enhanced cardiovascular risk management with targeted allied health [38]. Two retrospective cohort studies were undertaken between 2006 and 2014 using data from the Australian Government Department of Veterans Affairs, indicating a significant reduction in the risk of hospitalization (22%) for diabetes-related complications and a 23% reduction in the rate of potentially preventable hospitalization for heart failure related complications for patients who received GMPM [18, 49]. However, both studies were limited to Australian War Veterans aged 65 and over with congestive heart failure and diabetes.

There is no way of discovering whether patients receive additional allied health services, accessing them through outpatient clinics or private health insurance. CDM plans are currently supported by private health insurance [50]. Although private health insurance funds have been paying benefits for CDM plans for more than 10 years, evidence suggests insurers are struggling to expand their role in this area, such as identifying target groups and collaborating with other healthcare providers [51]. Most private health insurance funds have limited practice in primary care management and lack links with service providers [41]. Therefore, evidence for the effectiveness of CDM plans in private health insurance is limited.

While some study argue that technology can lead to an increased workload for GPs [45], some studies found that the application of eHealth tools such as cdmNet or Inca (Integrated Shared Care Planning Platform**)** for CDM planning in general practice settings has shown promising results [44, 52]. The use of eHealth tools can improve patient outcomes by providing a more coordinated approach to care, enhancing communication between healthcare providers, and enabling patients to take a more active role in managing their health [53]. This tool allows healthcare providers to access patient records, develop treatment plans, and monitor patient progress over time [52]. eHealth tools for CDM are challenging without a team-based approach as Vasi et al. [44] also suggested that a culture that values the participation of non-GP staff members fosters an environment where each member of the general practice team can contribute to CDM. This culture is essential for the successful integration of eHealth tools into a healthcare organization.

The importance of the CDM plan review to improve patients’ health outcomes was investigated in some studies. Although studies suggested that regular review is more critical than CDM plan preparation for complication prevention, the regular review is reported as either not occurring or only infrequently [33, 36, 38, 39, 42, 54]. Regular review every three or 6 months via GPMP and TCA review items is still much lower than the GPMP and TCA initiatives. There have been no GP long consultation claims for review via CDM plans suggesting that GPs do not use long consultations as a substitute for claim review [9]. The reasons behind this low usage are unclear. However, lack of awareness or understanding of the importance of CDM plan review among healthcare providers and patients could be the barrier. Additionally, the complexity of the review process may be perceived as time-consuming and challenging to implement in a busy primary care setting. Despite the lower usage of CDM plan review, it remains an important aspect of managing chronic conditions. Further research is needed to identify the barriers to its implementation and to develop strategies to increase its usage and effectiveness.

Medicare rebates for allied health services aim to provide some financial relief, but the likelihood of additional out-of-pocket costs remains a concern. Patients do not wish to pay out-of-pocket alongside their Medicare and private health insurance [40, 41]. This issue often leads to patients perceiving a dilemma when it comes to the value and necessity of allied health services, which can result in disparities in how they view their CDM plans [39]. This situation can further exacerbate disparities in the accessibility of healthcare services, particularly for people with lower incomes or those living in remote areas where healthcare services may be limited.

Foster and Mitchell [39] examined the different obligations in CDM and how they influenced healthcare professionals’ engagement with the recommended team care. Still, healthcare professionals often remained in authority over the patient, rather than sharing goal setting, decision-making, and responsibilities [33]. This can lead to a lack of patient engagement and a sense of disempowerment, which can negatively impact the effectiveness of CDM plans on their self-management. Additionally, healthcare professionals need to adopt a more patient-centred approach to care. This involves empowering patients to participate in goal setting and decision-making and sharing responsibilities for their self-management [55].

Outcomes from this review suggest person-centred care, shared responsibility, and a collaborative approach to CDM plans. All healthcare professionals involved in CDM plans need to reflect on how their primary healthcare settings may require changes for a particular population and lead to self-management support. This approach needs to be embedded at all levels to promote better integration of care and coordination.

### Review limitations

This review mainly focuses on current CDM plans in Australia because of the exclusive program rebated by Medicare, which provides a unique description of CDM plans in primary healthcare settings. Because of limited research on the effectiveness of CDM plans on diabetes self-management, other chronic conditions were considered. The lack of differentiation between different types of healthcare professionals in the study for CDM plans means that the study may have failed to account for important differences in roles, responsibilities, and experiences of different healthcare professionals involved in CDM. As a result, the findings may not accurately reflect the perspectives and experiences of all healthcare professionals involved in CDM, which may limit the relevance and applicability of the study’s findings. Therefore, it is important to consider the impact of such limitations when interpreting the findings of the study.

## Conclusion

This review highlights the importance of developing and utilizing effective CDM plans that support patient self-management. The effectiveness of CDM plans is influenced by various factors at three levels of patients, healthcare professionals, and system. Self-management support should be aligned with a patient-centred collaboration approach and shared decision-making but is not yet common practice. Therefore, understanding the key factors affecting patients’ self-management at different levels via CDM plans can be crucial to developing effective plans.

### Supplementary Information

**Supplementary Material 1.**

**Supplementary Material 2.**

## Data Availability

No datasets were generated or analysed for this scoping review.

## Abbreviations

CDM: Chronic Disease Management
GP: General Practitioner
GPMP: General Practitioner Management Plan
TCA: Team Care Arrangement
RACGP: Royal Australian College of General Practitioners
T2DM: Type 2 Diabetes Mellitus
HR-QoL: Health-Related, Quality of Life
CES: Central East Sydney
NSW: New South Wales
MBS: Medicare Benefits Schedule
eHealth: Electronic Health
PHI: Private Health Insurance
MoC: Model of Care
PBS: Pharmaceutical Benefits Scheme
CVD: Cardiovascular Disease
INCA: Integrated Shared Care Planning Platform
