# Supplementary material for: The effectiveness of chronic disease management planning on self-management among patients with diabetes at general practice settings in Australia: a scoping review
Source: BMC Prim Care. 2024 Mar 1;25:75. doi: 10.1186/s12875-024-02309-4 (PMC10905899; doi:10.1186/s12875-024-02309-4)
Supplement: Supplementary file 1 — Supplementary Material 1. [file 12875_2024_2309_MOESM1_ESM.docx]

**Appendix B: Full search strategy for all databases (Search Date undertaken May 2022)**

| Search strategy | search terms |
| --- | --- |
| **A**  **Population** | #1 “diabetes mellitus, type 2” OR DMII OR DM2 OR “type 2 diabetes” OR “diabetes mellitus type 2” OR NIDDM OR “impaired glucose tolerance”  #2 "chronic disease” OR " OR "noncommunicable diseases" OR "noncommunicable disease*" OR "NCD" OR "NCDs"  #3 "cardiovascular diseases" OR "myocardial infarction" OR "heart diseases" OR "heart failure" OR "cardiac disease*"  #4" cancer*" OR "neoplasms" OR "tumour*"    #5 "respiratory disease" OR "lung diseases" OR "respiratory illness" OR "respiratory condition" OR "pulmonary disease" OR "pulmonary disease, chronic obstructive” OR "pulmonary condition" OR "COPD" OR "asthma"  #6 "musculoskeletal disorder" OR "musculoskeletal diseases"  OR "musculoskeletal condition" OR "back pain" OR arthrosis" OR "osteoporosis" OR "osteoporosis" OR " rheumatoid arthritis"  #7 #1 OR #2 OR #3 OR #4 OR #5 OR #6 |
| **B**  **Outcome** | AND  # 8 “self-management” OR “patient self-management” OR “self-care” OR “patient self-care” OR “self-medication” OR self-administration” OR “blood glucose self-monitoring” OR “self-monitor”  #9 “patient-education” OR “behaviour therapy” OR” OR “patient-centred” Or Patient-centredness” OR “patient-focused” OR “patient empowerment” Or "lifestyle “ OR "lifestyle modification” OR "lifestyle change" OR “lifestyle adjustments” OR "health behaviour" OR "medication adherence" OR "treatment adherence” OR “treatment compliance " OR "patient preference” OR "patient participation” OR "treatment refusal" OR "disease management" OR "disease progression" OR "hospital admission" OR "patient readmission"  #10 #8 OR #9 |
| **C**  **Perception** | #11 "nurses" OR "nursing staff" OR practice nurses” OR "advanced practice nursing" OR "nurse practitioners" OR "nurse clinicians” OR “allied healthcare professionals” OR “allied healthcare services” OR “allied healthcare providers” OR” healthcare professional” OR “healthcare providers” OR “physiotherapist” OR “podiatry” OR “diabetes educator” OR “dietician”  #12 "factor “OR "constraint" OR "cause" OR "barrier" OR "facilitator" OR "enabler" OR "predictor” OR "outcome" OR "knowledge" OR "attitude" OR "perception"  #13 #11 OR #12 |
| **D**  **Intervention** | **#**14 “chronic disease management plan” OR “chronic disease management” OR “CDM” OR “CDM plan” OR “chronic care model” OR “chronic care plan” OR “Medicare-rebated chronic disease management plan” OR “Medicare-rebated CDM plan” OR “Medicare-subsidised chronic disease management plan” OR “Medicare-subsidised CDM plan” OR “general practice setting” OR “Primary care settings” OR “ medical clinic” OR “GP clinic” OR “superclinic”  # 15 “Shared decision-making process “OR “person-centred care approach” OR “Multidisciplinary team care approach” OR  “Interprofessional relationship” OR “collaborative approach”  #16 #14 OR #15 |

| **Database** | **Search** | **Result: number of hits** |
| --- | --- | --- |
| MEDLINE | Search #7 And #10 AND #13 And #16 | 89 |
| CINAHL | Search #7 And #10 AND #13 And #16 | 82 |
| PUBMED | Search #7 And #10 AND #13 And #16 | 76 |
| EMBASE | Search #7 And #10 AND #13 And #16 | 23 |
| Science Direct | Search #7 And #10 AND #13 And #16 | 10 |
| Wiley Online Library | Search #7 And #10 AND #13 And #16 | 30 |
| BMJ | Search #7 And #10 AND #13 And #16 | 39 |
| Google Scholar | Search 16 | 113 |
| EBSCO | Search #7 And #10 AND #13 And #16 | 28 |
| OVID | Search #17 And #10 AND #13 And #16 | 34 |
| Cochrane Library | Search #7 And #10 AND #13 And #16 | 15 |
| PshychiNFO | Search #7 And #10 AND #13 And #16 | 26 |
| Grey Literature | Search 16: specifically  “Medicare-rebated chronic disease management plan” OR “Medicare-rebated CDM plan” OR “Medicare-subsidised chronic disease management plan” OR “Medicare-subsidised CDM plan” | 12 |
