# Supplementary figures and images for: The effectiveness of chronic disease management planning on self-management among patients with diabetes at general practice settings in Australia: a scoping review
Source: BMC Prim Care. 2024 Mar 1;25:75. doi: 10.1186/s12875-024-02309-4 (PMC10905899; doi:10.1186/s12875-024-02309-4)

**Appendix 2: COVIDENCE draft data extraction form:**


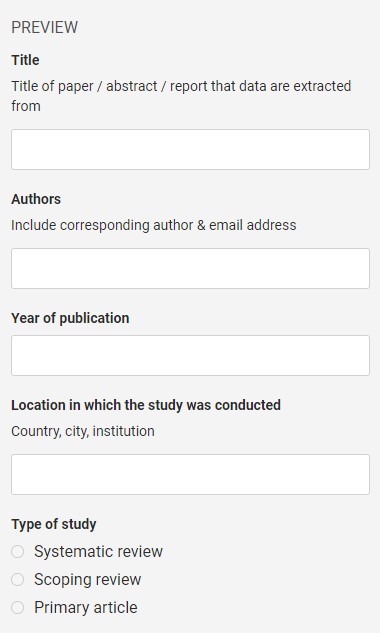


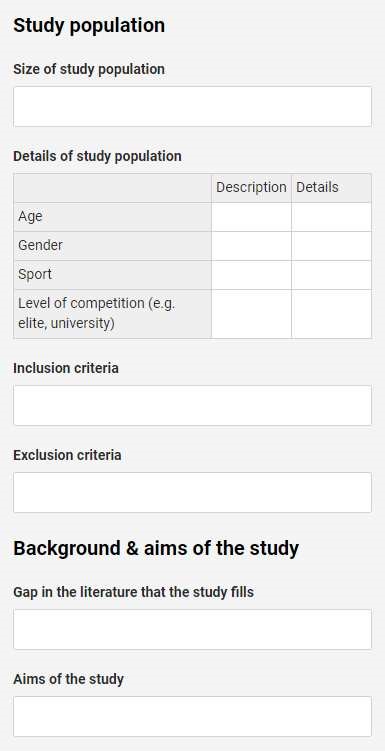


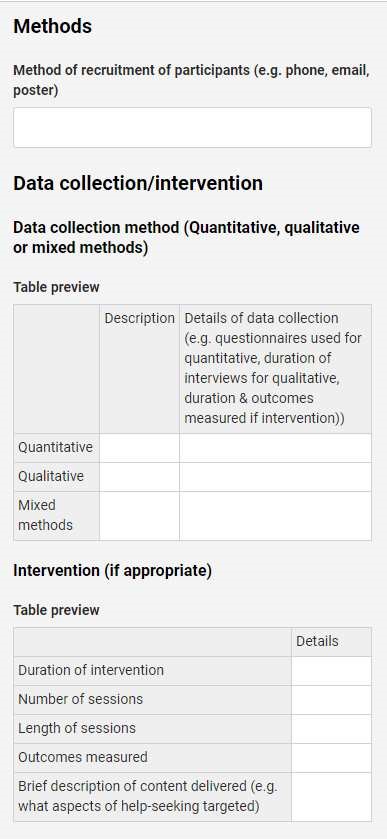


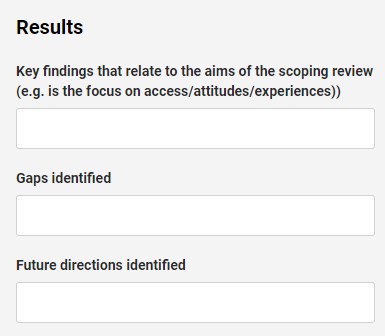

Supplement: Supplementary file 2 — Supplementary Material 2. [file 12875_2024_2309_MOESM2_ESM.docx]
